# Evaluating the current research landscape in gender-affirming surgery

**DOI:** 10.1016/j.jpra.2025.10.002

**Published:** 2025-10-17

**Authors:** Siyou Song, Zuivanna Rivas, Daniel Soroudi, Nina Mehta, Matthew F. McLaughlin, Alexis K. Gursky, Jasmin Wilson, Kiet T. Phong, Esther A. Kim

**Affiliations:** a Division of Plastic and Reconstructive Surgery, Department of Surgery, University of California, San Francisco, CA, USA; b School of Medicine, University of California, San Francisco, CA, USA; c School of Medicine, University of North Carolina, Chapel Hill, NC, USA; d Norton College of Medicine, SUNY Upstate Medical University, Syracuse, NY, USA; e Division of Plastic and Reconstructive Surgery, Department of Surgery, Icahn School of Medicine at Mount Sinai, New York, NY, USA; f Department of Bioengineering and Therapeutic Sciences, University of California, San Francisco, CA, USA

**Keywords:** Gender-affirming surgery, Research, Breast augmentation, Gender-affirming mastectomy, Metoidioplasty, Phalloplasty, Vaginoplasty

## Abstract

**Introduction:**

Research on gender-affirming surgery (GAS) is essential to advancing and optimizing surgical techniques, understanding long-term outcomes, and reducing health disparities. Multidisciplinary collaboration is crucial for optimal outcomes after GAS, but whether published studies reflect the multidisciplinary nature of clinical care is unknown. We sought to evaluate which specialties are publishing the most, the most published types of GAS, and the current trends in GAS research in order to elucidate the rapid growth of GAS.

**Methods:**

We conducted a cross-sectional study of all GAS research articles published on PubMed from March 2010, the year the Affordable Care Act was implemented, to 2022. For each article, we recorded senior author specialty, type of GAS investigated, year of publication, journal name, and funding source.

**Results:**

Of the 764 articles analyzed, the most were published on FFS (20.9 %) and vaginoplasty (20.6 %), with the fewest on metoidioplasty (2.88 %) (*p* < 0.01). There was an 86-fold increase in articles published from 2010 to 2022 (*p* < 0.01) and the number increased significantly for all GAS types except gender-affirming breast augmentation. Ninety percent of articles were not funded. Plastic surgeons produced the most publications on GAS (51 %), had the greatest increase of any surgical specialty (linear model slope = 0.18, *p* < 0.01), and produced the most articles on every type of GAS, with the exception of metoidioplasty, for which urologists published 81.8 % of articles.

**Conclusion:**

Research on GAS has increased significantly since the passage of the Affordable Care Act. Given that plastic surgeons published the most and had the greatest increase in publications amongst all specialties may reflect the fact that plastic surgeons comprise 79.5 % of gender-affirming surgeons in the United States. GAS is a rapidly expanding surgical field given its significant increase in publications over the past decade.

## Introduction

Gender-affirming surgery (GAS) encompasses a range of surgical procedures including those of the face, chest, and genitals. The incongruence between one’s gender identity and their assigned sex at birth can give rise to significant distress leading to the condition known as gender dysphoria.^1^^,^^2^ It is estimated there are over 1.3 million adults who identify as transgender and gender diverse;^3^ however, the approximate number of those with gender dysphoria is difficult to discern.^4^ The aim of GAS is to align a person’s gender identity to their ideal physical self and provides a form of treatment to mitigate gender dysphoria.^5^^,^^6^ Individuals who undergo GAS have lower rates of psychological distress, tobacco smoking, and suicidal ideation than those who do not undergo such surgeries.^7^

The number of GAS performed has increased over the years. A review of the incidence of GAS from the American College of Surgeons National Surgical Quality Improvement Program (ACS NQIP) found a substantial rise in GAS from 7 in 2010 to 1069 in 2018, reflecting a staggering 152-fold increase.^8^ Implementation of legislation that ensured gender-affirming care at the state or federal level, such as the Affordable Care Act (ACA) in 2010, resulted in a significant increase in the number of individuals receiving GAS, particularly among those with state-dependent insurance.^9^ As the number of GAS continues to rise, there has been a concurrent increase in the number of gender-affirming surgeons, with as many as 660 in the United States in 2019.^10^

Gender-affirming surgeries such as facial and genital masculinization or feminization, can be performed by surgeons specializing in the field of plastic surgery, urology, gynecology, otolaryngology (ENT), or oral maxillary facial surgery (OMFS).^6^ In the United States, plastic surgeons are the predominant surgeons performing GAS (79.5 %), with the second and third most common being otolaryngology (5.9 %) and urology (5.5 %), respectively.^10^ Research studies in GAS are essential to advancing and optimizing surgical techniques, understanding long-term outcomes, and reducing health disparities.

GAS is a rapidly expanding surgical field, yet there are few studies to evaluate the current trends in the research landscape of GAS. Multidisciplinary collaboration is crucial for good outcomes after GAS, but whether published studies reflect the multidisciplinary nature of clinical care is unknown, particularly the specialties actively researching in GAS. Given the multidisciplinary nature of GAS, collaboration among different specialties is crucial, and understanding the specific contributions of each specialty would be valuable. We therefore evaluated the trend of GAS publications over time, determined which specialties are actively researching and publishing on GAS, and assessed the types of GAS that are the focus of research. We hypothesized that plastic and reconstructive surgery would have a prominent role in advancing GAS research.

## Methods

### Study design

In this cross-sectional study of the current landscape of GAS research, we evaluated the trend in number of published studies on GAS by senior author specialty. Studies were identified on PubMed (NIH) from March 2010 to 2022. We chose March 2010 as the initial search date because it was the date that the ACA was passed.^11^ The ACA mandated insurance reimbursement for gender-affirming care, which contributed to an increase in GAS performed in the United States.^12^ Specialty categories include ENT, general surgery, OBGYN, OMFS, plastic surgery, urology, multidisciplinary, and other. The secondary outcomes were funding source, type of GAS, year of publication, and journal name. Funding sources included university/ academic, private, NIH/ government, industry, and no funding.

### Search method

We followed the methodology published by Mackenzie et al.^13^ Articles were found on PubMed using a Google Incognito session with cleared caches and cookies. We evaluated the following gender-affirming surgeries: facial feminization surgery, gender-affirming breast augmentation, gender-affirming mastectomy, metoidioplasty, phalloplasty, and vaginoplasty as these are the most common gender-affirming surgeries as described by the WPATH Standards of Care Version 8.^6^ The following GAS terms were searched: facial feminization surgery, gender-affirming mastectomy, gender-affirming breast augmentation, metoidioplasty, phalloplasty, and vaginoplasty. For masculinizing chest surgery, the following keywords were evaluated: “masculinizing chest surgery,” “gender-affirming mastectomy,” and “transgender mastectomy.” The keyword searches for breast augmentation were: “feminizing breast augmentation,” “gender-affirming breast augmentation,” and “transgender breast augmentation.” The keyword searches for metoidioplasty, phalloplasty, and vaginoplasty were: “gender-affirming metoidioplasty,” “gender-affirming phalloplasty,” and “gender-affirming vaginoplasty,” respectively. The keyword searches for FFS were: “facial feminization surgery” and “FFS.”

Articles were excluded if they were not related to GAS (including surgeries not related to gender-affirming care), published prior to March 2010, any duplicate from prior searches, reply articles, Y commentaries, guidelines, book chapters, had no available online abstract, were published in a non-English language, and/or were an animal/ basic science study.

### Data analysis

Each co-author involved in the study was assigned a set number of articles to review and code. For each article, the type of GAS evaluated, senior author specialty, funding type, and year of publication were recorded. An orientation meeting and practice round took place with all the coders to establish uniformity. If an author was unsure of how to code a category, then the first author and senior author reviewed the article and came to a consensus as to how it should be coded.

### Statistics

Additional metadata of the articles was retrieved from the PubMed database using the Entrez EFetch utility. Chi-square tests and chi-square posthoc tests were used to test for proportion of articles in each category.^14^ The Mann-Kendall test was used to verify publication trend over the years.^15^ Linear regression was used to fit a line for the number of publications over the years for each GAS type/senior author specialty. The Wilcoxon rank-sum test was used to compare the impact factor of journals publishing articles for each type of GAS. All statistical analyses were performed using R Statistical Software (v4.1.3) and R studio (release 3c53477a).

## Results

**Figure 1:**
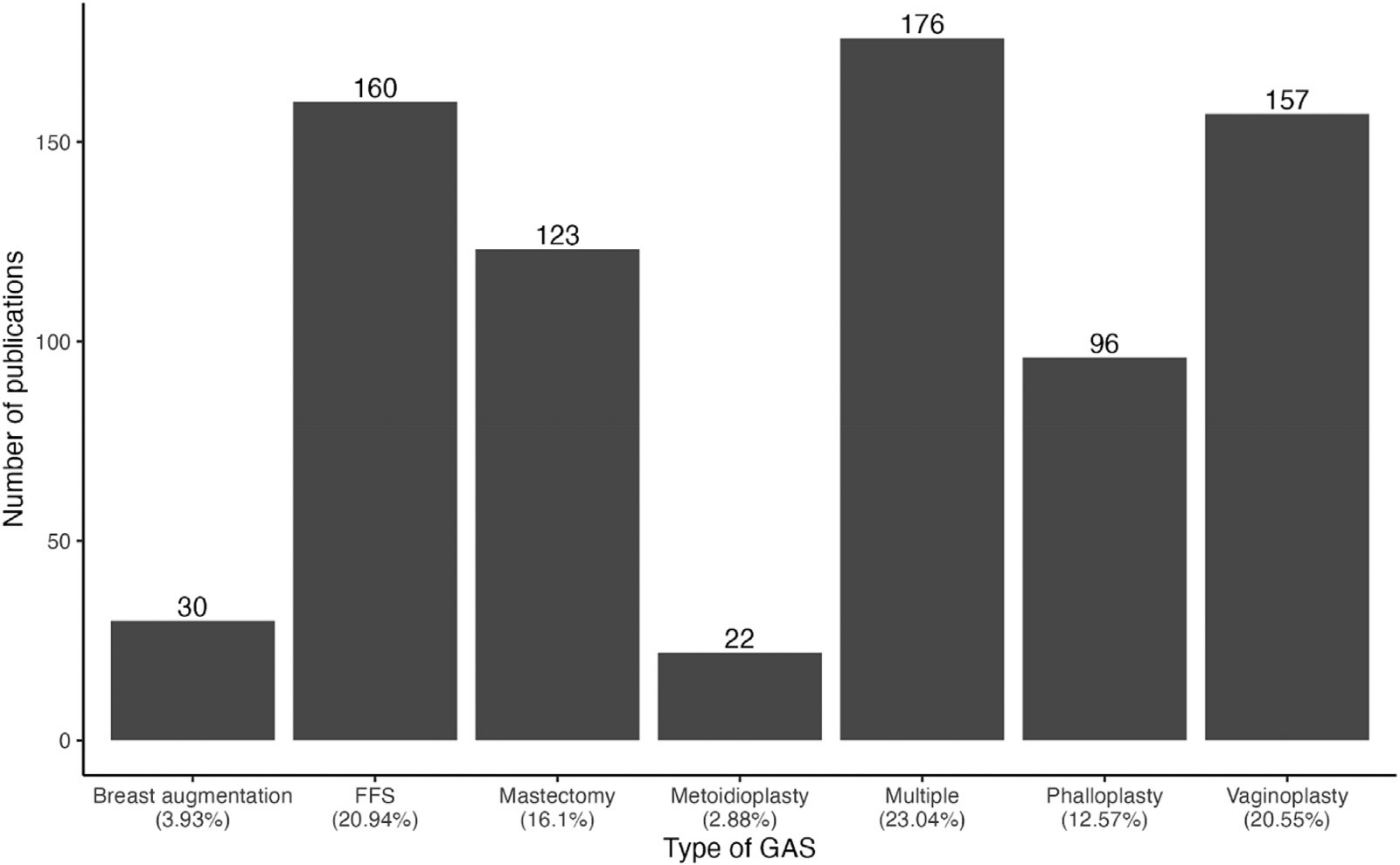
Number of publications categorized by type of gender affirming surgery published from 2010 to 2022. *p* < 0.01.

In total, 893 articles met our search inclusion criteria, of which 129 were duplicates, leaving 764 articles included in the final data analysis. Of the 764 articles, significantly more were published on multiple GAS types (23 %), FFS (20.9 %), and vaginoplasty (20.6 %), and the fewest were published on metoidioplasty (2.88 %) (*p* < 0.01, Figure 1).

**Figure 2:**
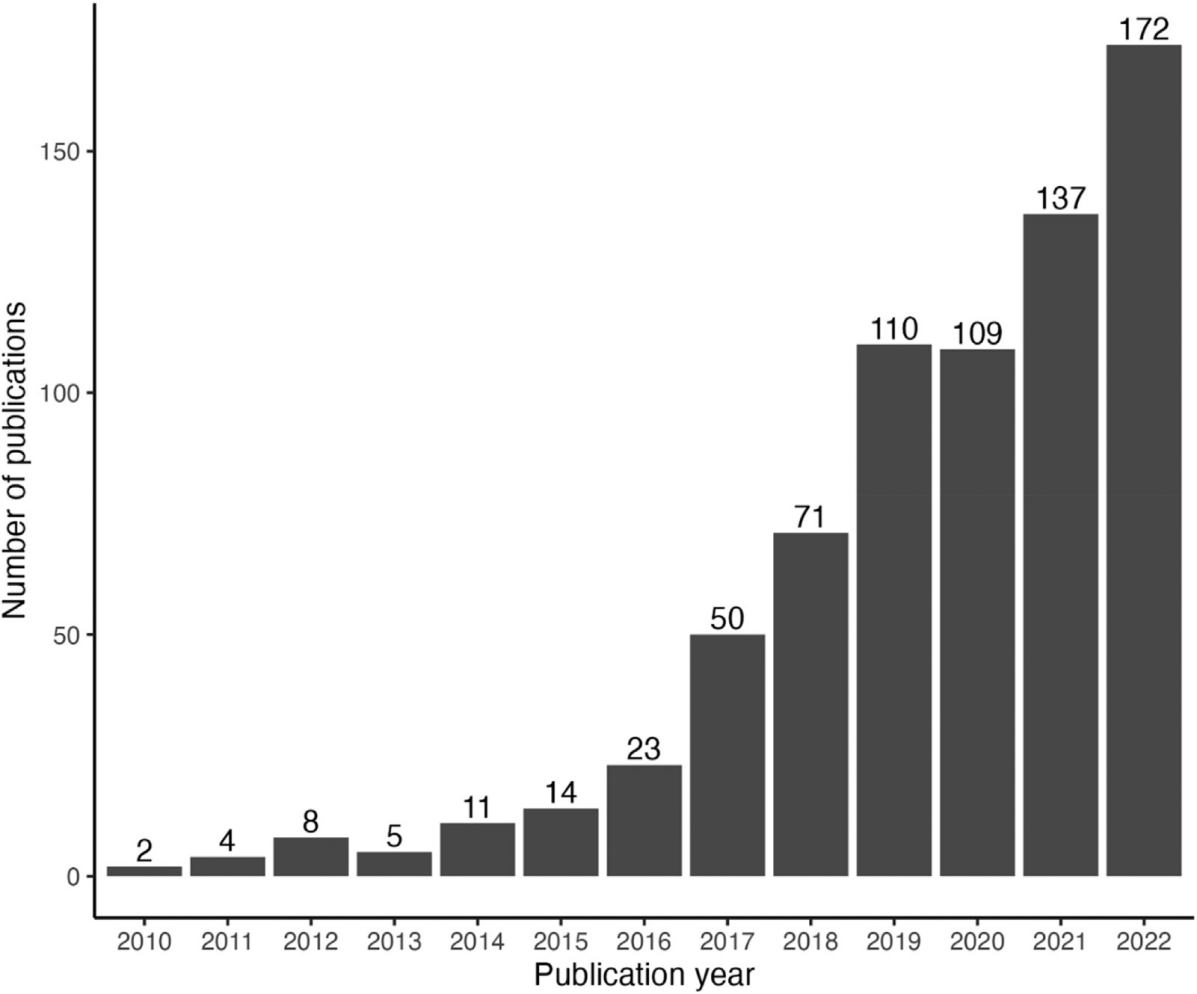
Number of publications on gender affirming surgery by year. *p* < 0.01.

**Figure 3:**
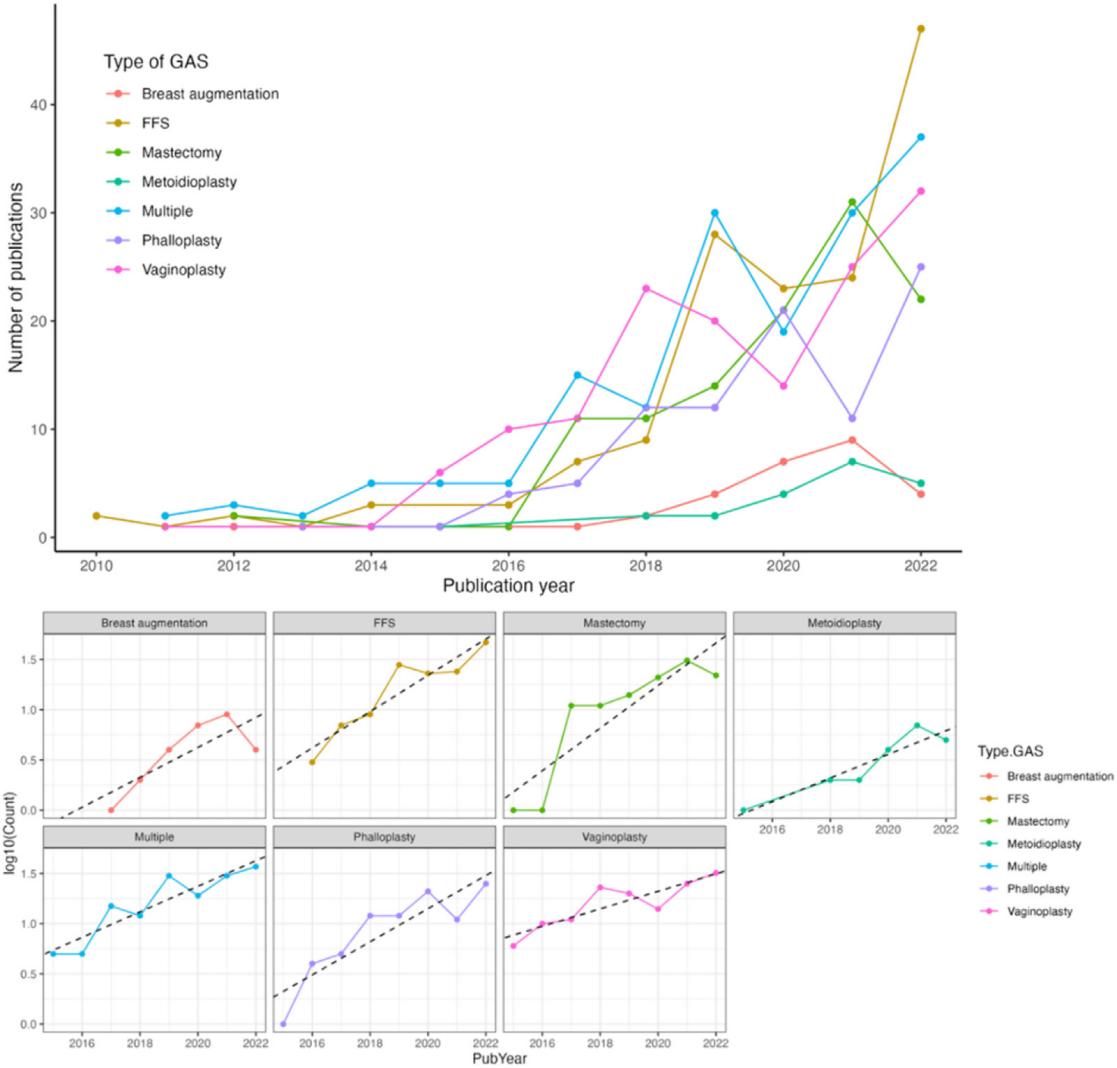
Number of publications on gender affirming surgery from 2010 to 2022. 3a. Number of publications by GAS type. *p* < 0.05. 3b Best fitting line for the increase of publications by GAS type from 2015 to 2022. *p* < 0.01 for a positive trend for each GAS type, except for breast augmentation (*p* = 0.06).

As expected, significantly more articles were published on GAS by the end of the 12-year period, with an 86-fold increase in the number of articles published per year (*p* < 0.01, Figure 2). For each type of GAS, the total number of yearly publications increased significantly from 2010 to 2020 (*p* < 0.05, Figure 3a). When a linear model was fitted for each GAS type, all types had significant increases in the volume of publications over the 12-year period (*p* < 0.01, Figure 3b), except for gender-affirming breast augmentation (*p* = 0.06, Figure 3b). The two GAS types that had the greatest increase in publications from 2010 to 2022 were phalloplasty (linear model slope=0.17) and gender-affirming mastectomy (linear model slope=0.17).

**Figure 4:**
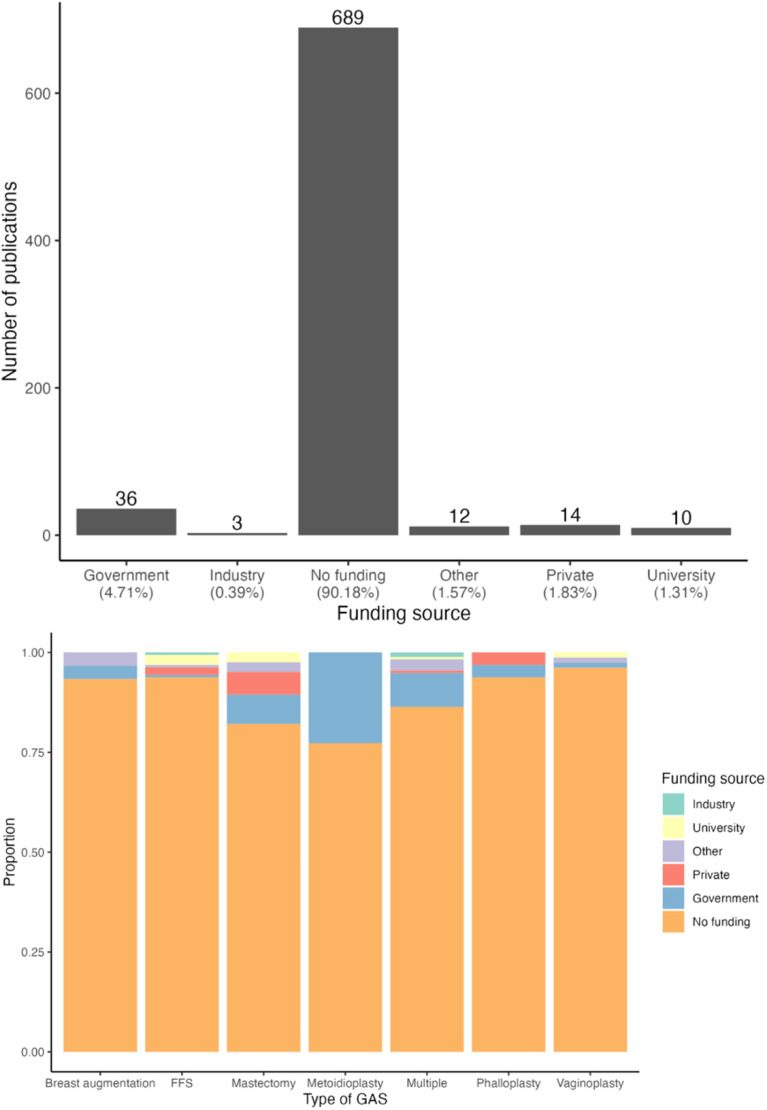
Type of funding. 4a. Number of publications categorized by type of funding. *p* < 0.01. 4b Proportion of funding source for publication in each type of gender affirming surgery. *p* < 0.01.

In terms of funding, 90 % of articles were not funded (*p* < 0.01, Figure 4a). This was largely true even when evaluated by GAS type, although 25 % of articles on metoidioplasty (five articles) received government funding (*p* < 0.01, Figure 4b).

**Figure 5:**
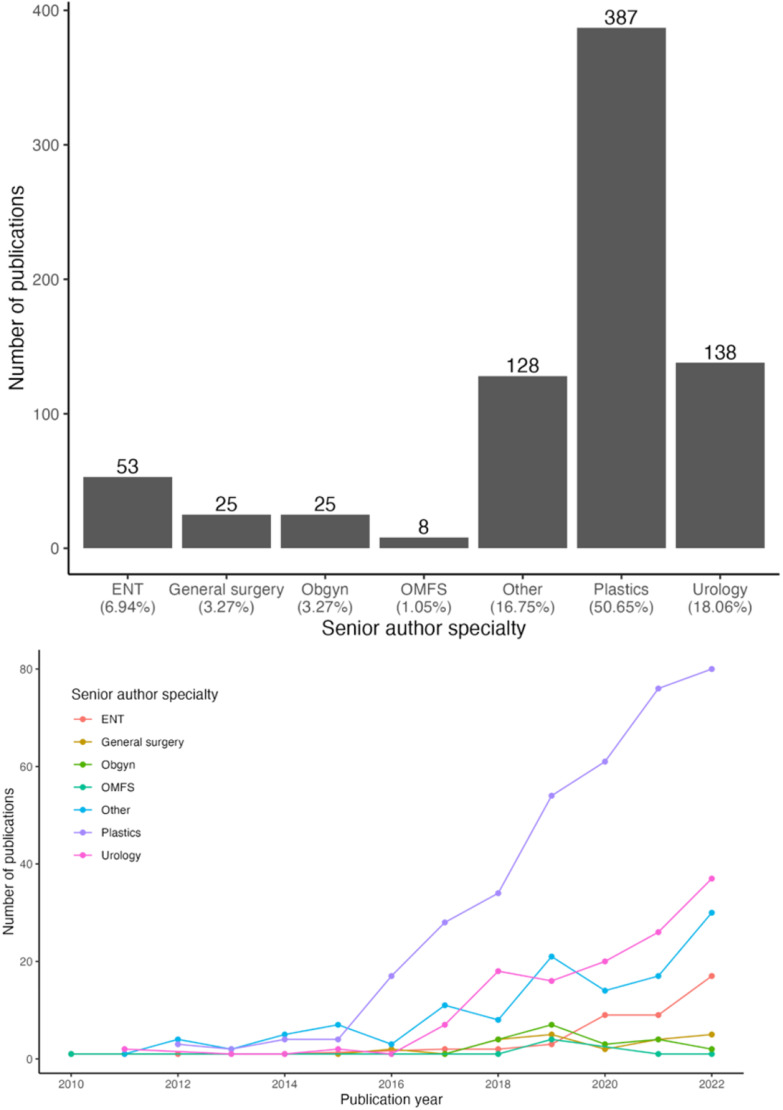
Number of publications by senior author specialty. 5a. Total number of publications by senior author specialty. *p* < 0.01. 5b Total number of publications by senior author specialty from 2010 to 2022. *p* < 0.01 for a positive trend except for general surgery (*p* = 0.055), OMFS (*p* = 1.00), and OBGYN (*p* = 1.00).

**Figure 6:**
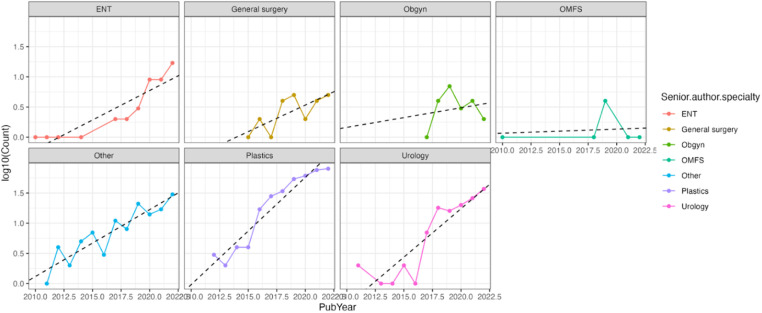
Best fit line for the increase in publications on GAS by senior author specialty. *p* < 0.01 for a positive trend for all specialties, except OBGYN (*p* = 0.69) and OMFS (*p* = 0.85).

**Figure 7:**
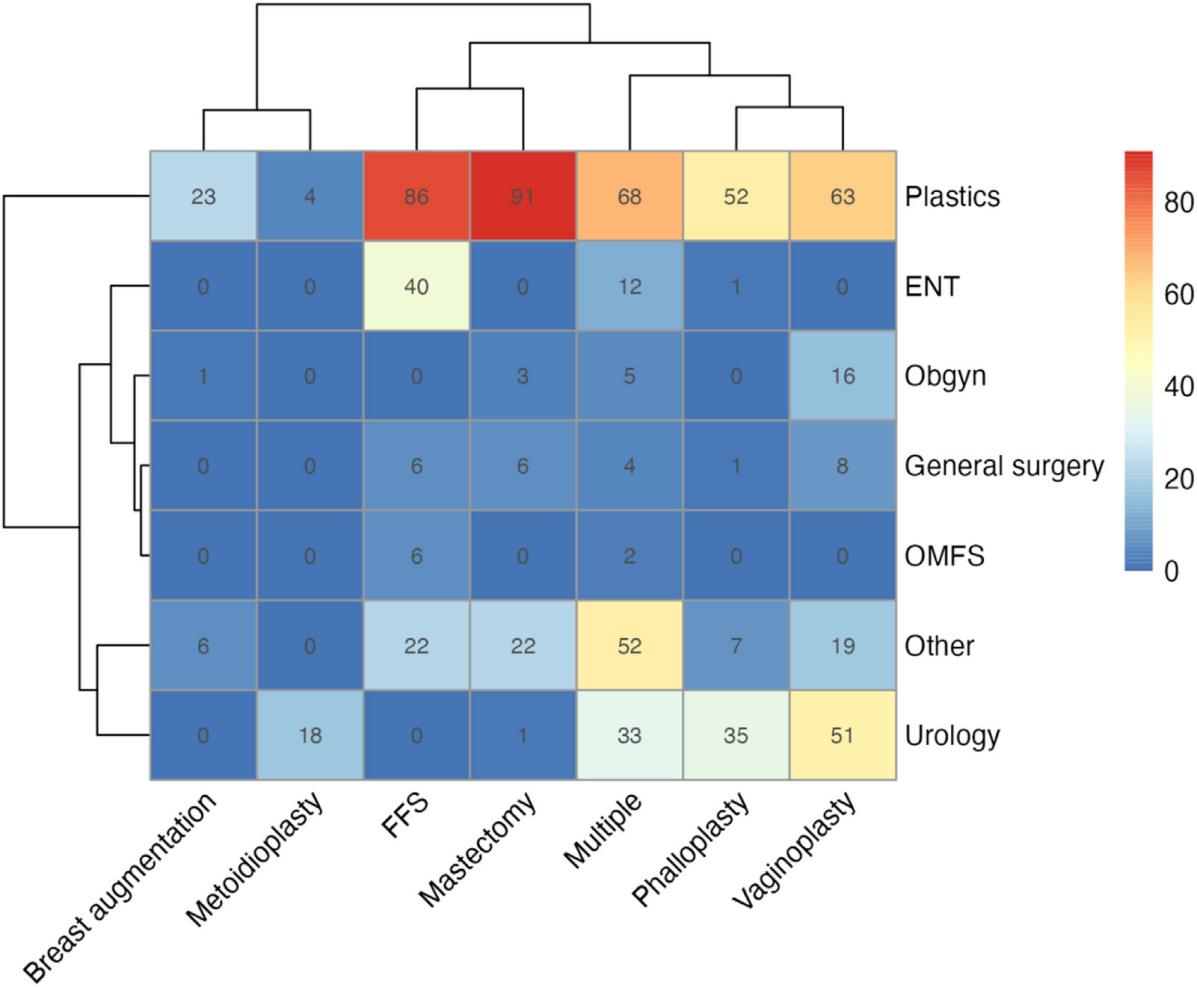
Number of publications by each GAS type by senior author specialty.

Analysis by senior author specialty showed that plastic surgeons produced the greatest number of publications on GAS (51 %) and OMFS produced the fewest (*p* < 0.01, Figure 5a). However, all specialties had significant increases in the number of GAS publications (*p* < 0.01), except for OMFS (*p* = 1.00) and OBGYN (*p* = 1.00) (Figure 5b). Over the 12-year study period, plastic surgeons also had the greatest increase in the number of publications as compared to any other surgical specialty (linear model slope=0.18, *p* < 0.01, Figure 6). The articles published by plastic surgeons mostly focused on gender-affirming mastectomy (23.5 %), FFS (22.2 %), and multiple types of GAS (17.6 %) (Figure 7). Plastic surgeons produced the most articles on every type of GAS, except for metoidioplasty, which urology published 81.8 % (Figure 7).

**Figure 8:**
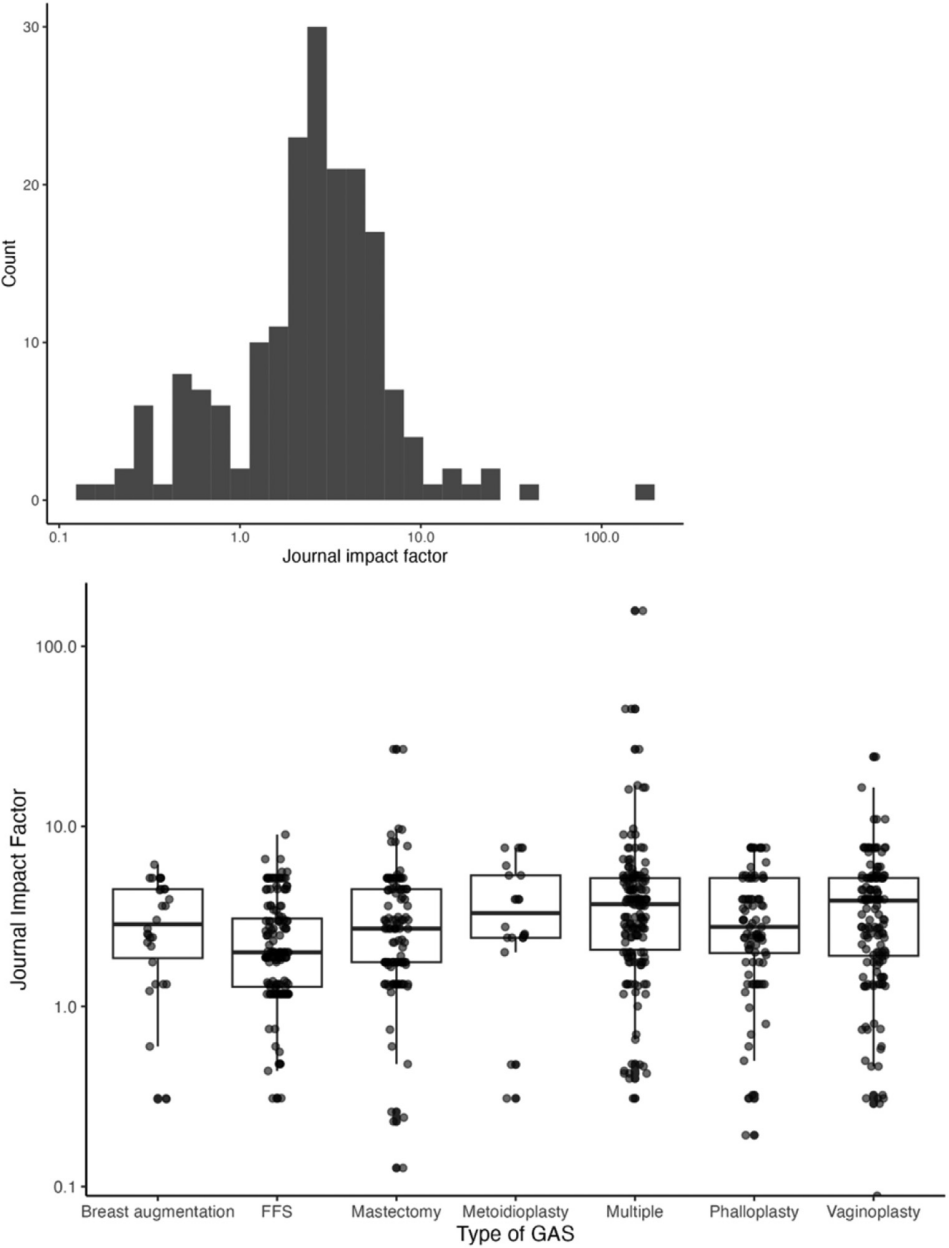
Impact factor. 8a. Distribution of journal impact factor for all the publications published from 2010 to 2022. 8b Distribution of journal impact factor by type of GAS for publications published from 2010 to 2022. *p* < 0.01. [*p*-value depicts difference in impact factor between FFS and Metoidioplasty, FFS and Multiple, FFS and Phalloplasty, and FFS and Vaginoplasty].

Seventy-five percent of articles were published in journals with an impact factor ranging from 1 to 10 (Figure 8a), even when evaluated by type of GAS (*p* < 0.01, Figure 8b).

## Discussion

This is the first study to evaluate the current research landscape for GAS by type of surgical specialty and type of GAS. Although the earliest case reports of GAS were published in the 1960s,^16^^,^^17^ GAS is a relatively new sub- surgical field that has grown exponentially in recent years.^18^ With the implementation of the ACA in 2010 and legislation prohibiting transgender-specific exclusions in insurance coverage in 2014,^12^^,^^19^^,^^20^ there was a three-fold increase in the number of patients seeking GAS just from 2012 to 2014, and a 155 % rise in GAS from 2016 to 2017.^21^^,^^22^ From 2016 to 2020, over 48,000 patients underwent GAS in the United States alone.^18^ Given the rapid increase in GAS performed in the United States, it is unsurprising that research published on GAS has similarly escalated. Compared to 2010, there has been an 86-fold increase in the number of research articles published in 2022 (Figure 2), reflecting current surgical trends and greater insurance coverage for GAS.

Our results show that plastic surgeons are the current leading contributors for research publications on GAS. Not only were plastic surgeons significantly publishing the most (50.7 %) on GAS over this 12-year period, but they also had the greatest increase in publications compared to other specialties (Figure 5). These findings are consistent with previous work that evaluated the top 50 most cited articles on GAS.^23^ The journal that published the most cited GAS articles (18 %) was a plastic surgery journal, and 38 % of the 50 most cited GAS articles were published in some type of plastic surgery journal.^23^

It is no surprise that plastic surgeons are leading contributors to research in GAS, since they comprise most of the gender-affirming surgeons in the United States.^10^ Of the 660 gender-affirming surgeons in 2019, 79.5 % were plastic surgeons, 5.9 % were ENT surgeons, and 5.5 % were urologists.^10^ Our results show that nearly 51 % of the GAS published articles were senior-authored by plastic surgeons, 18 % by urologists, and 7 % by ENT surgeons (Figure 5). With more plastic-surgery-trained gender-affirming surgeons, the trend we report will likely continue, with plastic surgeons producing the most research on GAS.

Overall, the fundamentals of plastic surgery align closely with the goals and surgical skills required for GAS. For example, when it comes to gender-affirming breast augmentation, plastic surgeons are highly trained and skilled in breast implant placement, postoperative care, and complications. This is reflected in the current publication landscape, with plastic surgeons publishing the greatest number of articles in gender-affirming breast augmentation compared to any other surgical specialty (Figure 7). Similarly, plastic surgeons are adept in microsurgery and tissue handling, which are necessary for free flap reconstruction in phalloplasty. Again, this is reflected in the research landscape where plastic surgeons are publishing the most on phalloplasty compared to any other surgical specialty (Figure 7).

As GAS continues to expand, publications on every type of GAS had significant increases over the 12-year period (Figure 3), except for gender-affirming breast augmentation (*p* = 0.06). Publications on gender-affirming breast augmentation may not have had a significant increase since these operations often entail similar techniques and approaches as breast augmentation for cisgender patients. Breast augmentation, including fat grafting, autologous reconstruction, and implant-based reconstruction, for cisgender patients has been routinely studied with over 8000 results on PubMed, with articles published as early as the 1950s.^24^^,^^25^ Although there are anatomic differences between cisgender and transgender female chests,^26^ there are still many similar operative and clinical decisions between cisgender and transgender breast augmentation procedures, such as operative approach, implant placement, postoperative care, and implant complications.

There are several limitations to this study. Only the senior author specialty was recorded; the various specialties listed in the authorship block were not recorded in our data collection for each study, hence this may not provide a complete and accurate representation of specialty collaboration. Additionally, only one engine search, PubMed, was utilized to search for articles; however, PubMed holds an extensive database with over 36 million citations and abstracts.^27^ Only articles published in English were included, which limits GAS research published in other languages.

Although plastic surgeons are currently leading contributors to GAS research, the field is constantly evolving and expanding. As that expansion continues as a result of greater insurance coverage and patient demand, we anticipate more research from all surgical specialties. Multidisciplinary collaboration in the operating room and in research will further optimize patient outcomes, improve surgical techniques and overall quality of life for transgender patients.

## Conclusion

This is the first study to evaluate the current publication landscape of GAS and elucidate the leading specialty contributors to the field. With the increase in gender-affirming surgeries and insurance coverage over the past decade in the U.S., research on GAS has expanded significantly. Plastic surgeons are currently the leading contributors to GAS research.

## Funding

None.

## Declaration of competing interest

None declared.
